# Huyang Yangkun formula regulates the mitochondria pathway of ovarian granulosa cell apoptosis through FTO/m6A-P53 pathway

**DOI:** 10.3389/fphar.2024.1491546

**Published:** 2024-11-08

**Authors:** Yang Li, Lingdi Wang, Jian Liu, Guangning Nie, Hongyan Yang

**Affiliations:** ^1^ Department of Gynaecology, The Second Clinical Medical College, Guangzhou University of Chinese Medicine, Guangzhou, China; ^2^ Department of Gynecology, The Affiliated Hospital of Shandong University of Traditional Chinese Medicine, Jinan, China; ^3^ Guangdong Provincial Key Laboratory of Clinical Research on Traditional Chinese Medicine Syndrome, Guangzhou, China

**Keywords:** m6Amethylation modification, premature ovarian insufficiency, traditional Chinese medicine, apoptosis, P53

## Abstract

**Background:**

Premature ovarian insufficiency (POI) presents a significant challenge to female reproductive health. The Huyang Yangkun Formula (HYF), a traditional Chinese medicinal formulation, has been utilized in clinical settings for the treatment of POI for over a decade. Nevertheless, the therapeutic application of HYF is considerably constrained by the lack of clarity regarding its underlying mechanism of action.

**Methods:**

The experimental procedures entailed administering VCD to female Sprague-Dawley rats at a dosage of 160 mg/kg/day over a period of 15 days, succeeded by a 100-day treatment with HYF. Blood serum samples were collected and analyzed using ELISA to quantify the concentrations of Anti-Müllerian Hormone (AMH), Follicle-Stimulating Hormone (FSH), and Estradiol (E2). The levels of N6-methyladenosine (m6A) were assessed through Dot blot analysis and liquid chromatography-tandem mass spectrometry (LC-MS/MS). Western blotting was employed to validate the differential expression of m6A-related catalytic enzymes and apoptosis-related regulators, including BCL-2, BCL-XL, and MCL-1, which may be implicated in the effects of HYF. Certain shRNA-COV434 cell line was constructed for the exploration of molecular mechanism, and then the potential targets were finally verified by MeRIP-qPCR.

**Results:**

HYF has been identified as having a significant influence on the development of residual ovarian follicles in rats with POI, especially during the initial stages. It was observed that HYF facilitates the progression of escaping antral follicles to full maturation. Additionally, HYF exhibited the capacity to enhance the proliferation of COV434, a human ovarian granulosa cell line, while concurrently inhibiting apoptosis within these cells. Notably, HYF treatment resulted in the downregulation of apoptotic proteins, including BCL-XL, cleaved-caspase 9, cleaved-caspase 3, and Bcl-2. Concurrently, m6A modification is implicated in the regulation of HYF. Both *in vitro* and *in vivo* studies indicate that FTO may play a role in the anti-apoptotic mechanisms mediated by m6A in ovarian granulosa cells influenced by HYF. Moreover, employing qPCR and MeRIP-qPCR techniques, P53 has been identified as the target gene for m6A modification mediated by FTO.

**Conclusion:**

These findings suggest that HYF holds promise as a potential treatment for POI and provide a more comprehensive understanding of the mechanism by which HYF operates, specifically its ability to prevent the BCL-2 mitochondrial apoptosis pathway mediated by P53 in ovarian granulosa cells of POI rats by regulating FTO/m6A-Tp53.

The results of this study indicate that HYF shows potential as a therapeutic intervention for primary ovarian insufficiency (POI) and contribute to a more thorough understanding of its underlying mechanisms. Specifically, HYF appears to inhibit the BCL-2 mitochondrial apoptosis pathway mediated by P53 in the ovarian granulosa cells of POI rats, through the regulation of the FTO/m6A-Tp53 axis.

## 1 Introduction

Premature ovarian insufficiency (POI) is a reproductive endocrine disorder, characterized by a cessation of function, which affects 3.7% of pregnant women under 40 years old (Welt, 2008; Golezar et al., 2019; Li et al., 2023). In addition to infertility, POI is associated with heightened risks of osteoporosis, cardiovascular disease, and premature mortality. This condition exhibits heterogeneity, with potential causes including autoimmune diseases, infections, or iatrogenic factors (Jiao et al., 2021; Ke et al., 2023). The etiology of approximately 80% of cases classified as ‘idiopathic POI’ remains elusive, indicating a lack of understanding regarding other biologically significant genetic factors contributing to POI (Liu et al., 2023).

In recent years, there has been a growing interest in exploring the involvement of N6-methyladenosine (m6A) regulation in diverse biological processes. Studies employing genetic loss-of-function approaches to investigate m6A methyltransferases, m6A-binding proteins, and m6A demethylases have underscored the crucial role of m6A modifications in governing gene expression during the onset and progression of reproductive development disorders. These disorders encompass various aspects, including sex differentiation, embryonic stem cell differentiation, and oocyte maturation (Lence et al., 2016; Sun et al., 2022). According to recent reports, an upregulation of m6A-modified transcripts has been observed during follicular recruitment or activation, which is regarded as a contributing factor to the dynamic expression of numerous genes associated with folliculogenesis (Hu et al., 2020; Yao et al., 2021). The role of m6A-methyltransferase, specifically methyltransferase-like 3 (METTL3), in facilitating the proliferation of ovarian granulosa cells has been well-documented (Hua et al., 2018). In a study conducted by Boxian Huang et al., the m6A RNA levels in granulosa cells from patients with premature ovarian failure (POF) infertility were examined, revealing an increase in m6A levels in these cells (Ding et al., 2018). A recent study by Liu et al. investigating transcriptome-wide N6-methyladenine (m6A) methylation in granulosa cells of women with diminished ovarian reserve has identified an increased number of m6A-methylated genes in the older age cohort (Liu et al., 2022). These findings indicate that the role of RNA m6A modification in ovarian aging remains inadequately understood and necessitates prompt and comprehensive further investigation.

Traditional Chinese medicine offers an effective treatment for premature ovarian insufficiency, especially for patients who cannot use hormone supplements or are hesitant due to concerns (Fu et al., 2022; Liu and Sun, 2023; Li et al., 2022). The Huyang Yangkun formula (HYF), derived from the traditional Chinese medicine recipe Danggui Buxue Tang, addresses ovarian aging symptoms in perimenopausal women. HYF consists of Astragali Radix, Herba Epimedii, Dioscoreae Rhizoma, Semen Cuscutae, Rehmanniae Radix, Angelicae Sinensis Radix, Glehniae Radix, at a ratio of 5:1:1:1:1:1:1. Based on the findings of our previous study, it has been observed that HYF treatment has demonstrated precise clinical efficacy in the management of POIs. Patients with declining ovarian function can potentially benefit from HYF therapy as it has the potential to enhance serum AMH and E2 levels, as well as regulate menstrual disorders (Li et al., 2020; Yafang, 2018). The depletion of follicles and subsequent ovarian aging can be attributed to abnormal apoptosis of oocytes or granulosa cells. Our previous research has indicated that the MAPK/P53/Bcl-2 family signaling pathways play a role in activating the apoptosis of ovarian granulosa cells (Yang et al., 2017). Building upon these findings, our research group has previously presented preliminary evidence suggesting that HYF treatment may facilitate follicular development through regulating Bcl-2 family-related mitochondrial apoptosis (Wang et al., 2021). Furthermore, it is postulated that the p53-mediated endogenous granulosa cell death pathway serves as the fundamental mechanism in the induction of follicular atresia. However, due to limited circumstances, the upstream mechanisms by which HYF regulates apoptosis have not been fully elucidated, warranting further investigation.

In this study, we developed an experimental methodology integrating both *in vivo* and *in vitro* approaches with two primary objectives: first, to elucidate the impact of m6A RNA modification on apoptosis in ovarian cells associated with primary ovarian insufficiency (POI); and second, to test our hypothesis that HYF modulates mitochondrial apoptosis in ovarian granulosa cells via the m6A/P53/BCL-2 signaling pathway. Furthermore, the study sought to identify specific targets through gene interference, thereby providing both experimental and theoretical foundations for the effective treatment of POI using HYF.

## 2 Materials and methods

### 2.1 Herbal materials and HYF extract preparation

Decoction of HYF, a Chinese herbal medicine prescribed at the clinic, obtained from Guangdong Kangmei pharmaceutical Company, Ltd (Guangdong, China), has been used to treat POI in Guangdong Provincial Hospital of Chinese Medicine for more than 10 years. HYF consists of seven botanical drug(s), including Astragali Radix, Herba Epimedii, Dioscoreae Rhizoma, Semen Cuscutae, Rehmanniae Radix, Angelicae Sinensis Radix, Glehniae Radix, at a ratio of 5:1:1:1:1:1:1 (Table1). After soaking for half an hour, HYF had been boiled for 1.5 h using a reflux extraction device. Then the extracted liquid was collected together, the liquid was concentrated using a rotary evaporator to a final concentration of 1.1 g⋅mL-1. This concentration of liquid is administered directly to animals by intragastric administration. The drug used *in vitro* cell assay is Freeze-dried, the freeze-drying conditions are: 40°C refrigeration, −20°C freezing for 2 h, −10°C freezing for 16 h, 20°C drying for 36 h, 35°C secondary drying for 36 h. Freeze-dried HYF were weighed and dissolved in F12 medium to a final concentration of 100 mg/mL and centrifuged at 14,000 rpm for 10 min, the supernatant was filtered with 0.22 μL filter before use.

**TABLE 1:** HYF composition.

English names (Chinese names)	Latin names	Botanical plant names	Dose
Milkvetch Root (huangqi)	Astragali Radix	*Astragalus didymophysus* Bunge	50 g
Epimedium (yinyanghuo)	Herba Epimedii	Epimedium brevicornu Maxim	10 g
Common Yam Rhizome (huaishanyao)	Dioscoreae Rhizoma	Dioscorea oppositifolia L	10 g
Dodder Seed (tusizi)	Semen Cuscutae	Cuscuta chinensis Lam	10 g
Rehmannia Glutinosa (shudihuang)	Rehmanniae Radix	ehmannia glutinosa (Gaertn.)	10 g
Chinese Angelica (danggui)	Angelicae Sinensis Radix	Angelica sinensis (Oliv.) Diels	10 g
Coastal Glehnia Root (beishashen)	Glehniae Radix	Glehnia littoralis F Schmidt ex Miq	10 g

### 2.2 Analysis of HYF by UPLC-MS/MS

Batch-to-batch consistency was monitored by quantifying marker compounds via UPLC-MS/MS analysis have been reported previously (Wang et al., 2021). Briefly, the chromatographic column was Thermo Hypersil GLOD C18 (2.1 × 100 mm, 1.9 μm). The mobile phase is 0.1% formic acid water (A) and 0.1% formic acid-acetonitrile mixture (D). Gradient elution: 0–2 min, 6% D; 2–25 min, 6%–95% D; 25–30 min, 95% D; 30–32 min, 95%–5% D. Flow rate 0.3 mL/min; Column temperature 40°C; Sample tray temperature 15°C; The sample size was 5 µL.

Mass spectrum conditions: HESI electrospray ion source; Scanning mode Full MS/dd MS2; Positive ion electrospray voltage 3.5 kV, negative ion electrospray voltage 3.2 kV; The sheath gas flow rate was 35 arb and the auxiliary gas flow rate was 10 arb. Capillary temperature 320°C; Probe heater temperature 350°C; Maximum spray current 100 A; S-Lens resolution 60; Scanning range m/z 100–1,500 Da; Quality resolution 70,000 (full width at half maxima, FWHM); Secondary resolution 17,500 FWHM.

### 2.3 Animal and experimental design

Animal studies were conducted following the guidelines approved by the Guangdong Provincial Hospital of Chinese Medicine Institutional Animal Care and Use Committee (Registration number: 2019032; Approval date: 15 July 2019). The animals used in this study were SPF-grade SD female rats provided by Guangdong Medical Laboratory Animal Center, License No. SCXK (Guangdong) 2019–0,035.

**FIGURE 1:**
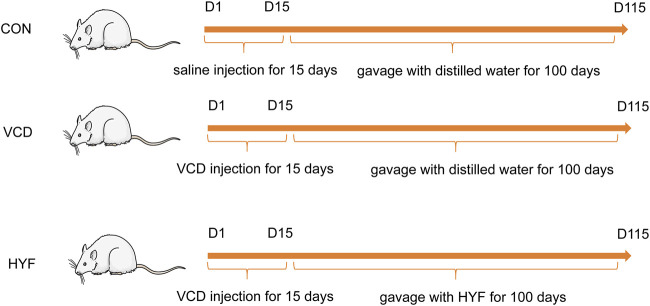
Overall animal experimental design.

The 28 days old SD rats were randomly divided into 3 groups using random numbers generated by computer (n = 7/group): normal control group (CON), VCD group (MOD) and Huyangyangkun formula group (HYF). Rats in VCD and HYF groups received intraperitoneal injection of VCD (Sigma Aldrich Korea, lot1302946) every day for 15 consecutive days (0.146 mL/kg) (Li et al., 2023). Then, the HYF group was gavaged with HYF (0.297 g/kg) for 100 days. Rats were finally euthanized using overdosed pentobarbital sodium and serum and ovaries were collected. Smears of rat vaginal exfoliated cells were taken between 9:00–10:00 a.m. daily (Figure 1).

### 2.4 HE staining and follicular count

To determine follicle numbers and observe follicular pathological alterations continuously, ovaries were fixed, embedded and sectioned as described before (Li et al., 2023; Nam et al., 2016). The section thickness was 5 μm, and the slices were successively sliced, and one slice was taken every 9 slices for HE staining. The sections of ovarian tissue stained by HE were observed under a microscope, and follicles were graded and counted according to a unified standard (reported previously), two pathologists performed blind numbers without knowing the grouping and took the mean of their readings for registration. The follicles were divided into 5 stages: primordial follicle, primary follicle, secondary follicle, antral follicle and mature follicle. To avoid the loss and duplication of counting, only the follicles in which oocytes can be observed are counted when counting small sinus follicles, sinus follicles, and mature follicles with larger diameters, and this is not necessary when counting small primary, primary, and secondary follicles.

### 2.5 Hormonal measurements

Blood was collected from orbital veins of rats at 30, 60, and 100 days after VCD injection for hormonal measurements. All rats were finally euthanized during the interestrous period, and blood was collected from abdominal aorta. Anti-Müllerian hormone (AMH), and estradiol (E2) were detected referring to the instructions of the ELISA kits (CSB-E11162r, CSB-E05110r, C0100090186, CUSABIO BIOTECH, Wuhan), follicle-stimulating hormone (FSH) were provided by (Elabscience, E-EL-R0391c, China).

### 2.6 Cell culture and plasmid construction

The COV434 line of immortalized granulosa cells were obtained from Sigma. Cells were grown at 37°C in a humidified 5% CO2 and 95% air and cultured in F12 medium (C11330500BT, Gibco, United States) containing 10% FBS (16000044, Gibco, United States) and 0.5% penicillin streptomycin sulfate (15140122, Gibco, United States). The granulosa cell apoptosis model was induced by VCD (Nam et al., 2016). Lentivirus construction was performed by Obio Technology (Shanghai, China). ShRNA interference fragment for Human FTO was designed and constructed into lentiviral vector by molecular biological means (pSLenti-U6-shRNA-CMV-EGFP-F2A-Puro-WPRE). The vector can be used to transfect cells to interfere with the expression of FTO gene in cells. pSLenti-CMV-MCS-3xFLAG-PGK-Puro-WPRE GL119 as a negative control (NC). Then, the COV434 cells were infected according to the optimal MOI value (MOI = 10), followed by screening for high purity infected COV434 cells.

### 2.7 RNA isolation and m6A quantification

Total RNA in ovaries and COV434 cells was extracted by Trizol reagent (Invitrogen, 15596026, United States) following the protocol. The mRNA was purified by Dynabeads^®^ mRNA Purification Kit (Thermo Fisher).

The purity of mRNA is measured by a NanoDrop method and diluted into different concentrations. For the rigor of the experiment, three experimental methods (ELISA, Dot blot and LC-MS/MS) were choosing to test the change in globa.

m6A levels in mRNA. For enzyme-labeled assay use m6A RNA Methylation Quantification Kit (Cat#ab185912, Abcam, United Kingdom), the RNA was incubated with m6A antibody in wells, and m6A levels were measured at 450 nm using a microplate reader. For Dot blot analysis and LC-MS/MS, the experimental process has been reported in detail in the previous article (Li et al., 2023), so we will not repeat it here.

### 2.8 MeRIP-qPCR

The methods for total RNA extraction, mRNA purification were described above, the amount and purity of total RNA were then controlled by NanoDrop ND-1000 (NanoDrop, Wilmington, DE, United States). The integrity of RNA was then detected by Bioanalyzer 2,100 (Agilent, CA, United States), and verified by agarose electrophoresis. The mRNA with PolyA was captured using the oligo (dT) magnetic beads (Dynabeads Oligo (dT) (No. 25–61005, Thermo Fisher, United States). Fragmentation was performed under high temperature conditions using the NEBNext^®^ Magnesium RNA Fragmentation Module (Cat#E6150S, United States) at 86°C for 7 min. Dynabeads Antibody Coupling Kit (Thermo Fisher, CA, United States) and m6A antibody (No. 202003, Synaptic Systems, Germany) was premixed in IP buffer (50 mM Tris-HCl, 750 mM NaCl and 0.5% Igepal CA-630). The IP product was synthesized into cDNA by Invitrogen SuperScript™ II Reverse Transcriptase (Cat#1896649, CA, United States). The resulting cDNA (including IP samples and Input samples) can be directly used for the next MeRIP-qPCR and conventional RT-qPCR.The sequences of the primers are shown in the (Supplementary Table 1).

The enrichment m6A of yap was expressed as the enrichment percentage relative to the input sample (%Input) = 2^−ΔΔCT^ (Input)-Ct (MeRIP)× Fd× 100%, where Fd is the input dilution factor (1/8).

### 2.9 Western blot

Cells and ovaries were harvested and lysed with 1x RIPA buffer (Beyotime, P0013B, China). Protein concentration was detected by Thermo Bicinchoninic acid (BCA, 23,227, United States) kit. Equal amounts of protein were dissolved in 5×loading buffer and separated in 10%–15% SDS polyacrylamide separation gels. The protein was transferred onto PVDF membranes (Beyotime, FFN10, China), then incubated with TBST solution containing 5% BSA for 1.5 h at room temperature and incubated with the primary antibodies at 4°C overnight. The next day, the membranes were washed and incubated for 1 hour with the secondary antibody. After the membranes were covered enhanced chemiluminescence (ECL), the images were shown by ChemiDoc XRS + chemiluminescence imaging system (Bio-rad). The results were analyzed with ImageJ software. Primary antibodies (all diluted at 1:1,000):ALKBH5, ab195377, Abcam; FTO, ab92821, Abcam; pJNK,4,668, CST; P38,8690, CST; PP38,4632, CST NRF2,16396-1-AP, Proteintech; BCL2,2876, CST; BCLXL, ab32370, Abcam; MCL1, ab32087, Abcam; BAX, 2,772, CST; BAK,12,105, CST; pBIM, ab17935, Abcam; PUMA,4,976, CST; Pro-caspase9/Cleaved-caspase9,10380-1-AP, Proteintech; Caspase3,9662, CST; Cleaved-caspase3, 9,664, CST; IgG,7,074, CST.

### 2.10 Statistical analysis

Data analysis was processed by SPSS 21.0 software. Mean ± Standard or Median (P25∼P75) was chosen to describe the data, depending on whether the data meet the normal distribution. *t*-test or Mann-Whitney U test was used to compare CON group with MOD group. One-way ANOVA or Kruskal–Wallis test was used for comparison of groups. *p* < 0.05 was considered statistically significant. And graphs were drawn by Graphpad Prism 8 software.

## 3 Results

### 3.1 Characterization of compounds in HYF

**FIGURE 2:**
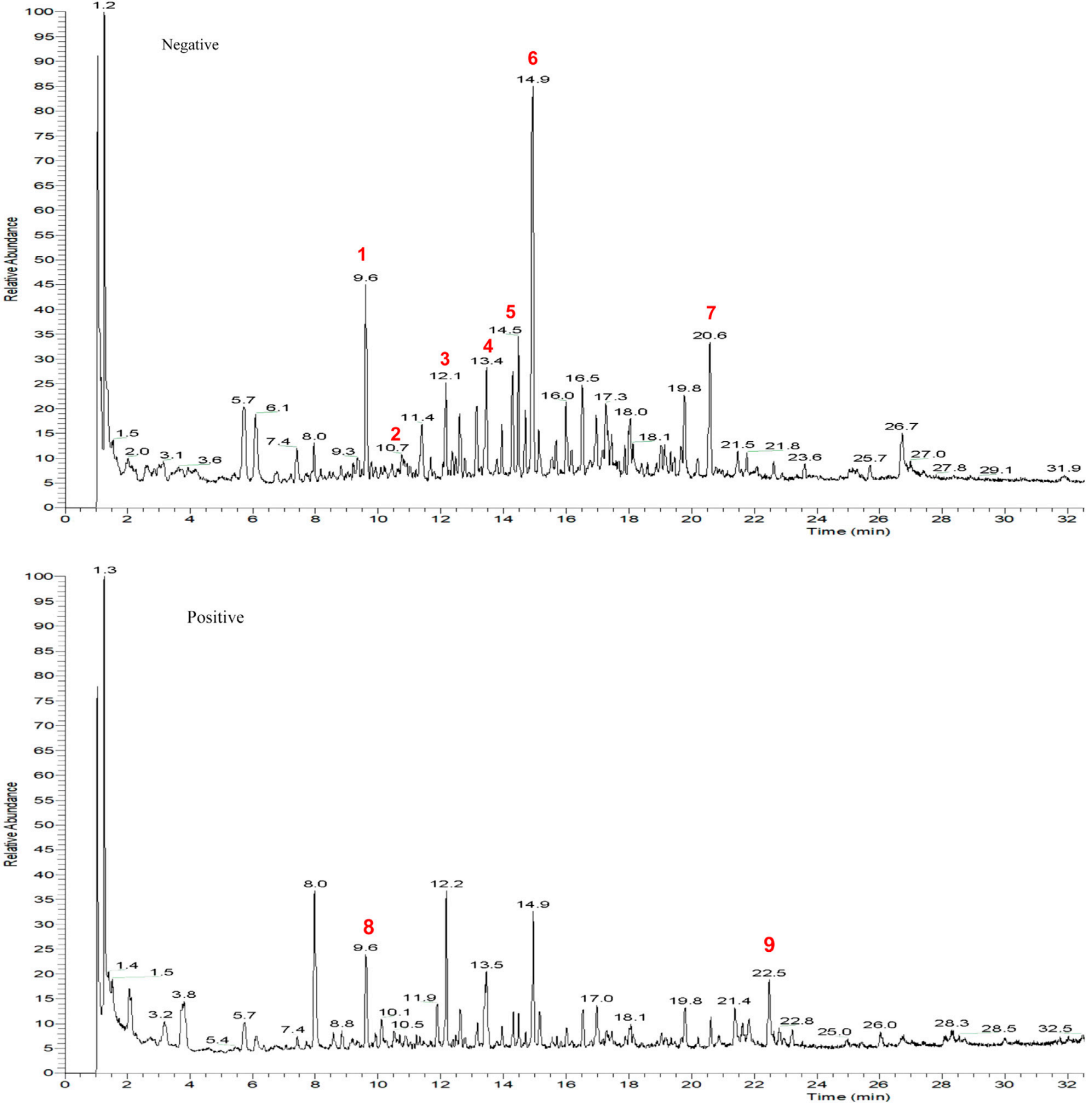
TIC (total ion chromatography) of HYF in negative and positive ion modes.1. Hyperoside, 2. Quercitrin, 3. Ononin, 4. calycosin, 5. epimedin B, 6, epimedin C, 7. baohuoside I, 8. ferulic acid, 9. Senkyunolide A.

Batch-to-batch consistency was monitored by quantifying marker compounds via UPLC-MS/MS analysis. Figure 2A and b display the quantification in negative and positive ion mode. UPLC-MS/MS was used to identify the active constituents of HYF. Nine major active components have been identified through the comparison of standard compounds (Table 2).

**TABLE 2:** The imformation of nine compounds identified in HYF by UPLC-MS/MS.

No.	Compound	t_R_ (min)	Formula	Observed Mass (m/z)	Detection mode (±)
1	Hyperoside	9.66	C_21_H_20_O_12_	463.08841	-
2	quercitrin	10.88	C_21_H_20_O_11_	447.09348	-
3	ononin	12.11	C_22_H_22_O_9_	475.12457	-
4	calycosin	13.44	C_16_H_12_O_5_	283.06122	-
5	epimedium B	14.50	C_38_H_48_O_19_	853.27679	-
6	epimedium C	14.79	C_39_H_50_O_19_	867.29297	-
7	baohuoside I	20.60	C_27_H_30_O_10_	513.17688	-
8	ferulic acid	9.16	C_10_H_10_O_4_	193.04996	+
9	senkyunolide A	20.12	C19H21ClN2O3S	193.12166	+

### 3.2 Protective effect of Huyang Yangkun formula on ovarian function in premature ovarian insufficiency (POI) rats

**FIGURE 3:**
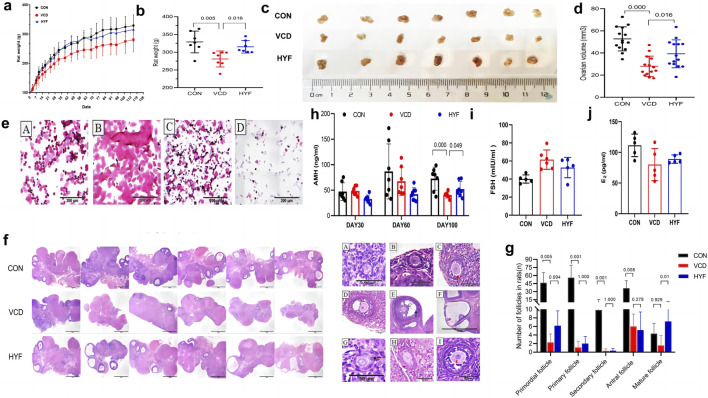
Protective effect of HYF on ovarian function in POI rats **(A, B)** Body weight changes in rats (a. Body weight record, b. Final body weight; n = 7; Compared with CON group, ^##^ *p* < 0.01; Compared with VCD group, ^*^ *p* < 0.05) **(C, D)** Comparison of ovary weight and volume in each group (c. Images of ovaries of rat. d. Ovarian volume; n = 7). **(E)** Representative images of estrous cycle of rats (A. Proestrus, B. Estrus, C. Metestrus, D. Diestrus). **(F)** Morphological changes of ovarian tissue in rats (n = 6, maximum transverse section, HE, ×40, scale bar = 1 mm). **(G)** Number of primordial follicles, primary follicles, secondary follicles, antral follicles and Mature follicles in each group. Data are shown as the mean ± SD, n = 6 **(H, J)** Detection of sex hormone in rats (AMH, FSH and E2, n = 6).

Ovarian function can be predicted by assessing the estrous cycle, follicular development, and hormonal fluctuations. Rats were periodically weighed throughout the experimental period. It was observed that the body weight of rats in the VCD and HYF groups increased at a slower rate than that of the CON group, with this difference becoming evident by the first month of VCD modeling (Day 28). The body weight of rats in the VCD group was found to be lower than that of the CON group (*p* < 0.01), while the HYF group exhibited higher body weight than the VCD group (*p* < 0.05) (Figures 3A, B). The ovarian structures of the rats are depicted in Figure 3C. Bilateral ovaries from each rat were subjected to weight measurement, and their dimensions were recorded, specifically the long diameter (L, in millimeters) and the short diameter (S, mm). The ovarian volume was subsequently calculated using formula V = L *S^2^/2, mm3). When compared to the control (CON) group, the VCD group demonstrated a significant reduction in ovarian volume, amounting to 46.89%. In contrast, the group not exposed to VCD exhibited a significant increase in ovarian volume by 39.28% relative to the VCD group (Figure 3D). To evaluate alterations in the estrous cycle, we performed an analysis of vaginal cell shedding at 30, 60, and 100 days following HYF administration, in addition to daily sampling over a period of 10 consecutive days. The VCD model rats exhibited significant disruptions in their estrous cycle, which intensified with prolonged VCD exposure. Similarly, the HYF group experienced disturbances in their estrous cycle; however, some individuals in the HYF group maintained regular estrous cycles by the conclusion of the experiment (Figure 3E; Supplementary Table 2). Figure 3F illustrates the maximum section of pathological sections of the ovary in three groups of rats. The VCD group exhibited significantly lower numbers of follicles at all stages compared to the CON group (*p* < 0.01, *p* < 0.01, *p* < 0.01, and *p* < 0.05, respectively). Following HYF intervention, there was a significant increase in the number of original and mature follicles compared to the VCD group (*p* < 0.05, *p* < 0.01, respectively, Figure 3G).

Serum anti-Müllerian hormone (AMH) levels were employed as a sensitive biomarker for assessing ovarian reserve, with serum samples collected on days 30, 60, and 100 following VCD injection. No significant differences in AMH levels were observed among the three groups at 30 and 60 days post-injection. However, at 100 days post-injection, the serum AMH level in the VCD group was significantly reduced compared to the control (CON) group (*p* < 0.01, Figure 3H). Furthermore, the HYF group demonstrated a significant upward trend in AMH levels 100 days post-VCD injection compared to the VCD group (*p* < 0.05, Figure 3I). Blood samples for follicle-stimulating hormone (FSH) and estradiol (E2) were collected in accordance with the estrous cycle of rats (4–5 days), and a sex hormone cycle line chart was developed to observe their respective trends. The FSH line chart indicates that the CON group is consistent with previous literature (Olvera-Juárez et al., 2020), whereas the line chart for the VCD group exhibits a completely irregular pattern (Figure 3J and Supplementary Figure 1). The E2 line chart indicates that the rats in the normal control group display periodic fluctuations, aligning with previous findings by Olvera-Juárez et al. (2020), in contrast, the E2 line chart for the VCD group exhibits a disordered pattern, characterized by the absence of wave crests or the emergence of multiple peaks. Meanwhile, the HYF group shows a slight degree of regularity, evidenced by the presence of wave crests. Furthermore, a comparison of baseline E2 levels revealed no statistically significant differences among the three groups (Figure 3J and Supplementary Figure 1).

### 3.3 The anti-apoptotic effect of Huyang Yangkun formula is related to jnk/P53/mitochondrial apoptotic pathway

**FIGURE 4:**
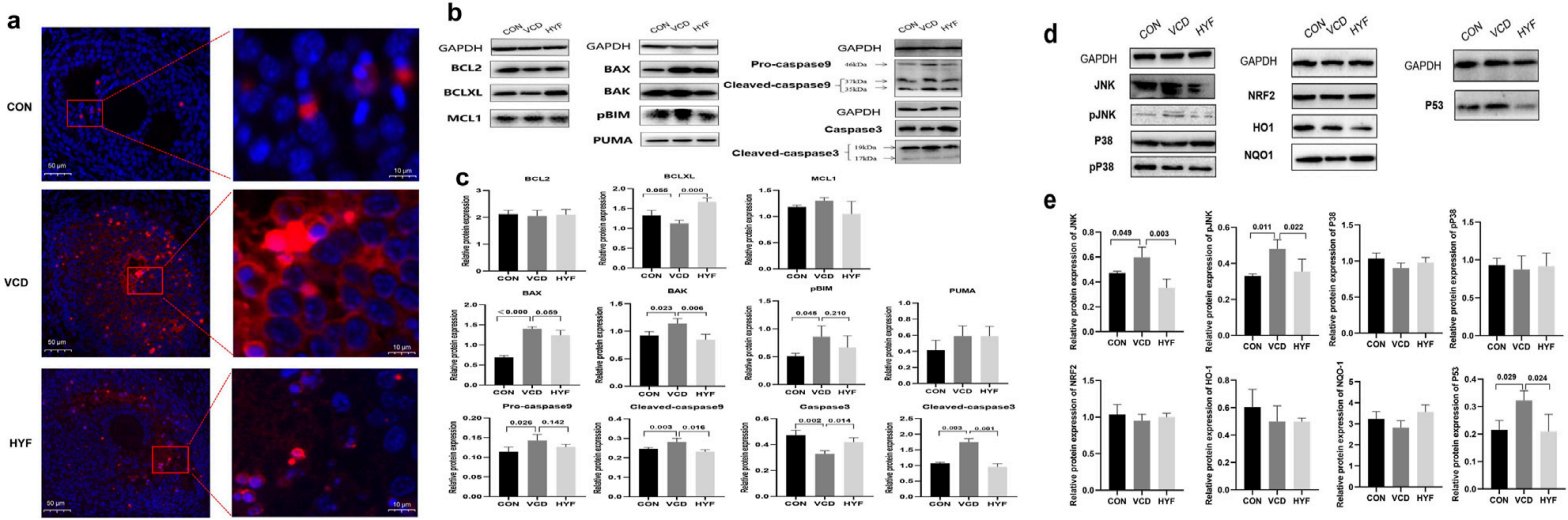
HYF exhibited protective effects against VCD-induced apoptosis. **(A)** The apoptosis of ovarian tissue was detected by immunofluorescence. **(B, C)** Western blot analysis of the expression of BCL-2 family and Caspases proteins in rat ovarian tissues (n = 3). **(D, E)** Western blot analysis of protein expression of potential target in rat ovarian tissue (n = 3).

TUNEL staining was first used to detect the apoptosis of ovarian granulosa cells in the CON, VCD and HYF groups. As shown in Figure 4A, CON showed less apoptosis signals to the naked eye, while the ovaries in the VCD-induced POI group showed a lot of apoptosis signals. On the contrary, granulosa cell apoptosis in POI rats decreased after HYF intervention. Meanwhile, according to this result and previous research basis, we detected the expression levels of various anti-apoptotic proteins associated with both extrinsic and intrinsic by WB experiment.

TUNEL staining was initially employed to assess apoptosis in ovarian granulosa cells across the CON, VCD, and HYF groups. In Figure 4A, the CON group exhibited minimal apoptotic signals visible to the naked eye, whereas the VCD-induced POI group demonstrated a substantial increase in apoptotic signals. Conversely, granulosa cell apoptosis in POI rats was reduced following HYF intervention. Based on these findings and previous research, we conducted Western blot (WB) experiments to evaluate the expression levels of various anti-apoptotic proteins associated with both extrinsic and intrinsic pathways.

The results of the rat ovarian tissue test revealed that in the VCD group, there was an increase in the expressions of pro-apoptotic proteins BAX, BAK, and pBIM (*p* < 0.01, *p* < 0.05 and *p* < 0.05, respectively), as well as an increase in the expression of Caspase9 protein in both the precursor Pro-caspase9 and Cleaved caspase9 forms (*p* < 0.05). Additionally, the expression of Cleaved caspase3 was also found to be upregulated (*p* < 0.01). In contrast, the HYF group exhibited an increase in the anti-apoptotic protein BCLXL in the BCL-2 family (*p* < 0.01), a decrease in the pro-apoptotic protein BAK (*p* < 0.01), and a decrease in both Cleaved-caspase9 and Cleaved-caspase3, when compared to the VCD group (*p* < 0.01, Figures 4B, C).

To explore the potential upstream molecular targets of HYF anti-mitochondrial apoptosis, based on the analysis of relevant literature and preliminary research, we established a potential pool of upstream signals consisting of eight potential targets, namely, JNK, pJNK, P38, pP38, NRF2, HO1, NQO1, and P53. These signals were investigated as potential regulators of the mitochondrial apoptosis pathway.

The results indicated that compared to the control group, the expressions of JNK, pJNK, and P53 were significantly increased in the VCD group (*p* < 0.05), while no statistically significant differences were observed for P38, pP38, NRF2, HO1, and NQO1. In comparison to the VCD group, the expression levels of JNK, pJNK, and P53 were significantly downregulated (*p* < 0.01, *p* < 0.05, and *p* < 0.05, respectively), while no statistically significant differences were observed for P38, pP38, NRF2, and HO1 proteins (Figures 4D, E). Our research findings indicate that HYF has a significant inhibitory effect on the expression of P53 protein under VCD stimulation. This suggests that the P53 signal may have a crucial role in the molecular mechanism of HYF. Furthermore, the anti-apoptotic effect of HYF is partially mediated through the JNK in the MAPK kinase system.

### 3.4 mRNA m6A methylation plays a role in the mechanisms underlying VCD-induced POI and HYF

**FIGURE 5:**
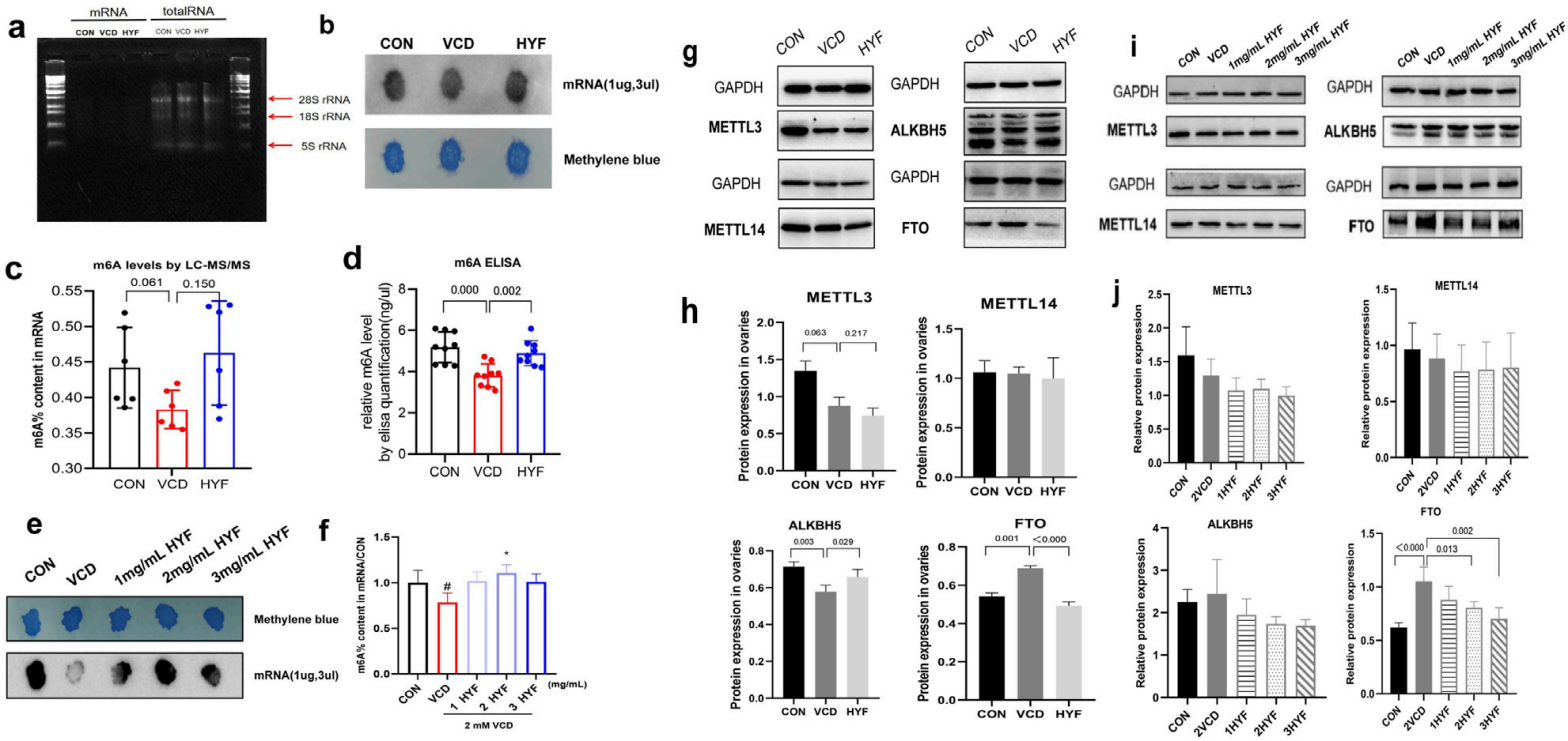
Effect of HYF on m6A levels and the expression of methylase *in vivo* and *in vitro*. **(A)** The quality of mRNA extraction was assessed through RNA electrophoresis. **(B)** Rat ovary m6A levels were determined by Dot Blot **(C, D)** Rat ovary m6A levels were determined by m6A ELISA and LC-MS/MS **(E, F)** M6A levels in cov434 cells were determined by Dot Blot and LC-MS/MS **(G, H)** Comparison of the expression levels of m6A related catalytic enzymes in ovary of rats (n = 3) **(I, J)** Comparison of m6A methylase expression levels *in vitro* COV434 cells (n = 3).

Evidence suggests that m6A modifications play a significant role in the reduction of ovarian follicular reserve. This study also explores this aspect by extracting RNA from rat ovarian tissues. The quality of the mRNA extraction was evaluated using RNA electrophoresis, as illustrated in Figure 5A. The levels of m6A-RNA modification in rat ovarian tissues were assessed utilizing ELISA, dot blot, and LC-MS/MS methodologies. The results from all three detection techniques demonstrated a consistent trend, revealing that the m6A modification in the VCD group was significantly reduced compared to the CON group (*p* < 0.01). Furthermore, the global m6A level in the HYF group was found to be elevated relative to the VCD group (*p* < 0.01, Figures 5B–D). It is noteworthy that the m6A modification of mRNA in the VCD group demonstrated a reduction of 15.36% relative to the CON group, whereas the HYF group exhibited an increase of 20.71% in m6A modification. Furthermore, to investigate the protective role of m6A involvement in HYF on ovarian granulosa cells, human ovarian granulosa cells (COV434) were employed for relevant analyses. The m6A-RNA modification level in the *in vitro* experiment was evaluated using Dot Blot, which revealed a significant reduction in the m6A modification level in the VCD group compared to the CON group (Figures 5E, F).

In this study, the protein expression levels of key m6A catalytic enzymes, specifically METTL3, METTL14, ALKBH5, and FTO, were analyzed to assess potential alterations in these enzymes within the ovaries of rats across three distinct groups. The expression levels of METTL3 and METTL14 exhibited no significant differences among the groups. Conversely, the expression of the demethylase ALKBH5 was significantly reduced in the VCD group (*p* < 0.01) and significantly elevated in the HYF group (*p* < 0.05). Additionally, the expression of FTO, another demethylase, was significantly increased in the VCD group and significantly decreased in the HYF group (*p* < 0.01, Figures 5G, H). The results indicate possible modifications in m6A-related catalytic enzymes in the ovaries of rats across various experimental groups. Western blot analysis of *in vitro* cells demonstrated a statistically significant difference in FTO expression between the groups, with an F-value of 8.239 and a *p*-value of 0.003 (Figures 5I, J). In contrast, no significant differences were observed in the expression levels of METTL14, ALKBH5, and METTL3 among the groups. This section of the study provides additional evidence that modifications in m6A are intricately linked to the fate of ovarian cells. Furthermore, it clarifies the role of the FTO/m6A axis in the regulatory effects of HYF on ovarian granulosa cells.

### 3.5 The FTO/m6A-Tp53 pathway is involved in the anti-apoptotic effect of HYF: In vivo POI rat model or *in vitro* VCD-cov434 cell apoptosis model

Previous findings suggested a significant link between FTO/m6A RNA methylation and targets like pJNK, P53, PUMA, and BAX at the protein level, both *in vivo* and *in vitro*. To explore if FTO/m6A, influenced by HYF, is related to the mitochondrial apoptosis pathway triggered by these targets, we conducted two experiments: verifying functional proteins after FTO knockdown *in vitro*, and examining m6A modified transcripts in ovarian samples.

### 3.6 (1) FTO mediate m6A modification to participate in the anti-apoptosis molecular mechanism of HYF

**FIGURE 6:**
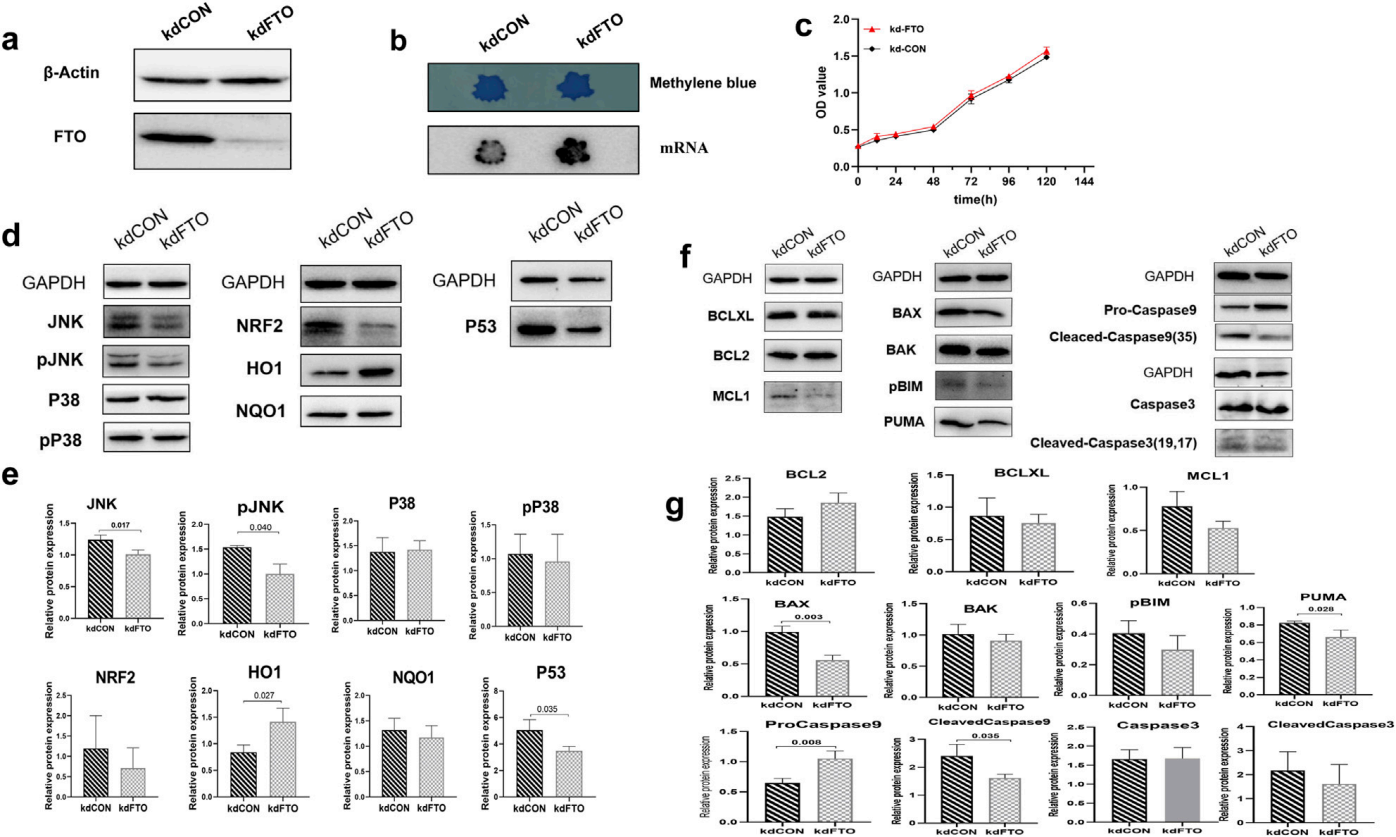
FTO mediate m6A modification to participate in the anti-apoptosis molecular mechanism of HYF **(A, B)** Effect of FTO gene knock-down on FTO protein expression and m6A level in ovarian granulosa cells. **(C)** Effect of FTO gene knockdown on ovarian granulosa cell proliferation **(D, E)** Protein changes of ovarian granulosa cell candidate target pool after FTO knockdown, n = 3; Compared with kdCON group **(F, G)** Expression changes of BCL-2 family and Caspases protein in FTO knockdown granulosa cells (n = 3).

As shown in Figure 6A, the expression of the FTO protein was significantly reduced in the FTO gene knockdown group compared to the blank control group. Consistent with previous reports, FTO knockdown resulted in a significant increase in the level of m6A (Figure 6B). The proliferation and apoptosis of COV434 cells were not affected by FTO knockdown (Figure 6C). Although FTO knockdown did not ultimately lead to significant changes in apoptosis phenotype, some changes occurred in certain proteins inside the cell, it was observed that the expression of JNK, pJNK, and P53 significantly decreased following FTO knockdown (*p* < 0.05, Figures 6D, E). Likewise, the protein levels of BAX, PUMA, and Cleaved-caspase9 were also diminished upon FTO knockdown (*p* < 0.01, *p* < 0.05, *p* < 0.05, respectively. Figures 6F, G).

### 3.7 m6A-Tp53may be the pathway through which m6A modification participates in the anti-apoptotic effect of HYF

**FIGURE 7:**
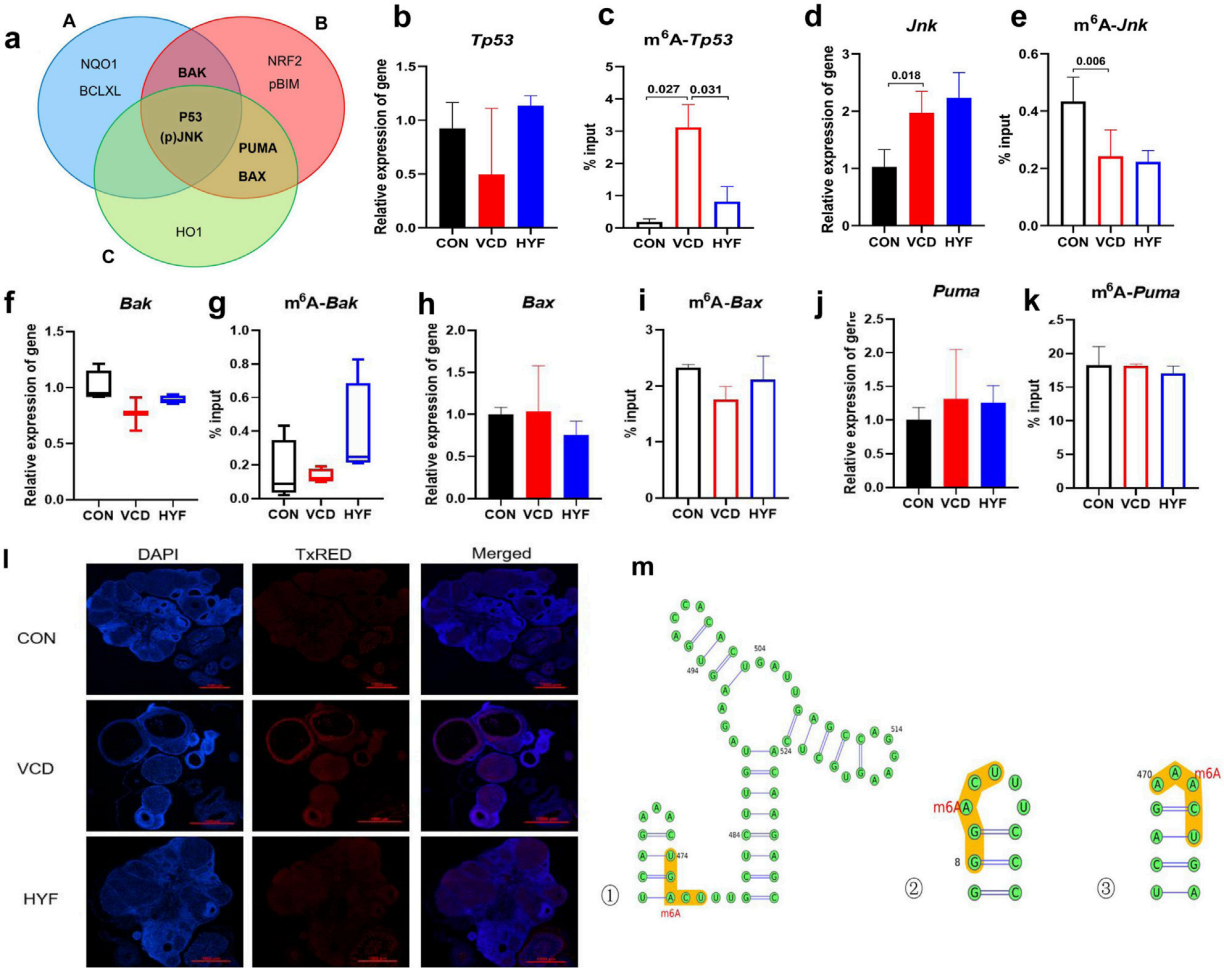
The pathways of m6A-Tp53 involved in the anti-apoptotic effect of HYF **(A)** Venn diagram shows the screening results of major differential proteins (A. Differential proteins in animal experiments, B. VCD model cell experiments, C. FTO knockout cell differential proteins **(B–K)** Enrichment of m6A-modified mRNA in ovarian tissue, analyzed by MeRIP-qPCR, the percentage of the input is shown (n = 3–4; b-c. Tp53 and m6A-Tp53; d-e.Jnk and m6A-Jnk; f-g.Bak and m6A-Bak; h-i. Bax and m6A-Bax; j-k. Puma and m6A-Puma). **(I)** Immunofluorescence detection of P53 in rat ovarian tissue **(M)** Prediction of m6A modification sites on Tp53 transcripts (top three confidence levels).

The potential target proteins were screened and identified through both *in vivo* and *in vitro* experiments, with the results being descriptively summarized (Figure 7A). Our findings led to the identification of five potential targets: m6A-TP53, m6A-JNK, m6A-PUMA, m6A-Bax, and m6A-Bak, which are hypothesized to exert significant influence. To validate this hypothesis, we performed preliminary validation experiments on these five core candidate targets using rat ovarian tissue. These experiments specifically examined the differences between groups of total transcripts and m6A-modified transcripts.

As illustrated in Figure 7B, the mRNA expression of Tp53 demonstrated a decreasing trend in the VCD group and an increasing trend in the HYF group, which was contrary to the expression pattern observed for the P53 protein. This result is of considerable interest. Notably, the expression of m6A-Tp53 significantly increased in the VCD group and significantly decreased in the HYF group (Figure 7C, *p* < 0.05). This observation provides a potential explanation for the elevated levels of P53 protein in the HYF group. Moreover, Jnk expression was observed to be upregulated in the VCD group, while no statistically significant difference was detected between the HYF and VCD groups. In contrast, the expression of m6A-Jnk was downregulated in the VCD group, with no significant difference between the HYF and VCD groups (Figures 7D, E). As for Puma, Bax, and BAK, the results demonstrated minimal variation across the three groups, with inconsistent expression levels noted at both the protein and gene expression levels. Moreover, the abundance of m6A-modified transcripts did not correspond to the previously discussed proteins (Figures 7F–K). In conclusion, m6A modifications on Jnk and Tp53 (m6A-JNK, m6A-TP53) may play a contributory role in the pathogenesis of POI, with the regulation of m6A-TP53 potentially having a more significant impact on the anti-apoptotic effects of HYF.

The current study offers preliminary evidence indicating the involvement of m6A-Tp53 in the pharmacodynamic mechanism of HYF *in vivo*. To further investigate this, we performed immunofluorescent staining to assess P53 expression and localization in rat ovaries (Figure 7L). Our results demonstrated a reduced fluorescence intensity in the CON and HYF groups, while a significant increase was noted in the VCD group. Additionally, the fluorescence signals were primarily localized within the follicular granulosa cells, suggesting that the observed alterations in P53 and m6A-Tp53 within the ovary likely originate from these cells.

In addition, the SRAMP (http://www.cuilab.cn/sramp) and RMBase v2.0 databases (http://rna.sysu.edu.cn/rmbase) were employed to forecast the methylation locations within the mRNA sequence. The m6A modification sites pertaining to p53 were primarily located near the initiation sites of gene transcription. A total of eight methylation sites were identified in association with P53, with one discovery exhibiting a very high level of confidence, three discoveries demonstrating high confidence, four discoveries indicating moderate confidence, and five discoveries suggesting low confidence (Figure 7M; Supplementary Table 3; Supplementary Figure 2).

## 4 Discussion

Premature ovarian insufficiency (POI) is a key cause of female infertility due to early ovarian decline. Currently, no drug can restore POI-related reproductive function, and diminished ovarian function is considered irreversible. However, traditional Chinese medicine shows promise in treating menstrual issues and aiding conception (Li et al., 2022). According to the principles of traditional Chinese medicine, HYF exhibits significant potential in the treatment of POI (Yafang, 2018). VCD is an effective method for creating POI mouse models through continuous intraperitoneal injection of 160 mg/(kgd) VCD for 15 days. Rodent ovaries exhibit POI-like clinical characteristics approximately 70 days after stopping the injections. We utilize these POI models to study HYF’s efficacy and mechanism, having previously explored different Chinese medicine intervention times (25, 50, 90, and 105 days). By evaluating follicle development, estrus cycle, and serum sex hormones, we found that ovarian function improved more at 90 and 105 days after HYF treatment compared to 50 and 25 days. Our study indicates that VCD-induced POI causes irreversible ovarian damage without natural repair, but early HYF treatment can partially preserve ovarian function. Given these findings, our aim was to elucidate the underlying mechanisms and examine the potential therapeutic implications for POI.

From a clinical standpoint, anti-Müllerian hormone (AMH) is extensively acknowledged as a dependable and sensitive indicator for evaluating ovarian reserve, owing to its minimal variability across the menstrual cycle (Anderson et al., 2022; Nelson et al., 2023). In our study, it was observed that the administration of HYF significantly increased serum AMH levels (*p* < 0.05, Figure 3H), suggesting a potential protective effect on ovarian reserve function. Furthermore, the follicle count results demonstrated a trend towards an increased number of primary follicles and pre-antral follicles under the influence of HYF, while a slight decrease was observed in the number of antral follicles compared to the VCD-POI group (Figure 3G). This finding seems to contradict the observed elevation of AMH levels in the HYF group, a phenomenon of significant interest. It is well-established that AMH is synthesized by the granulosa cells of primary, preantral, and small antral follicles. Thus, it is puzzling why AMH levels are elevated despite the lack of a substantial increase in the number of antral follicles. This suggests that two potential factors may influence AMH secretion: the number of granulosa cells and their capacity to produce AMH (Alvaro Mercadal et al., 2015; Desongnis et al., 2021). It is reasonable to hypothesize that in individuals with primary ovarian insufficiency (POI), the basal antral follicles, which contain competent granulosa cells, represent the genuinely viable and effective follicles capable of maturing. In the current study, the administration of HYF led to a significant increase in mature follicles in rat models. Therefore, it can be inferred that HYF may facilitate follicular development by enhancing the competence of granulosa cells within the follicles (Supplementary Figure 3).

Prior research on the role of m6A in ovarian aging disorders has been relatively limited. In a pertinent study by Jiang et al. (2021), a comparative analysis was performed on cumulus granulosa cells from older women with diminished ovarian response (≥37 years old) and younger women with normal ovarian response (<37 years old). The study revealed that increased m6A modification levels were exclusively associated with a decrease in FTO protein, whereas other m6A-related catalytic enzymes did not show significant changes. The m6A RNA levels in granulosa cells from patients with primary ovarian insufficiency (POI) were investigated by Boxian Huang and colleagues (Ding et al., 2018; Huang et al., 2019), who reported an elevation in m6A levels within these cells. Similarly, increased m6A RNA modification levels were observed in CTX-induced POI ICR mice compared to normal ICR mice. However, this finding is inconsistent with our results, which indicate that the decrease in ovarian m6A levels in POI rats induced by VCD may be attributed to the downregulation of the methyltransferase METTL3, coupled with the upregulation of the demethylase FTO. In our earlier study, we analyzed m6A modification levels in C57 mouse ovaries at different developmental stages: 4 weeks (puberty), 7 weeks (sexual maturity), 9 weeks (body maturity), and 24 weeks (middle and old age). We found that m6A levels increased from puberty to sexual maturity but gradually decreased from sexual maturity to middle and old age (Supplementary Figure 4). The results indicate a pattern of m6A modification in the mouse ovary, with levels rising during reproductive development and decreasing with age. Various experiments suggest that these modifications are influenced by factors like cellular origins, model inductions, and detection methods. In summary, the effects of RNA m6A modification and its catalytic enzymes on ovarian aging are still emerging, requiring further study due to varied outcomes.

The apoptotic function of P53 is stringently regulated, and in quiescent, non-stimulated cells, P53 exists as a minimally stable protein with a relatively short half-life of approximately 30 min. However, when stress-induced damage exceeds the cell’s repair capacity, P53 adopts a lethal role by initiating cellular apoptosis (Meulmeester and Jochemsen, 2008; Wu and Deng, 2002). This study explores the key role of the mitochondrial apoptosis signaling pathway and its link to upstream protein signals. We identified and detected candidate proteins *in vitro* and *in vivo*, focusing on core differential proteins, VCD exposure raised ovarian P53 protein levels, activating apoptotic proteins like BAX and BAK and increasing follicle apoptosis. However, TP53 mRNA levels were downregulated. We proposed that P53 undergoes post-transcriptional modifications, the discovered that VCD-induced apoptosis is caused by the upregulation of demethylase FTO, which results in the upregulation of m6A-Tp53 transcripts, which in turn leads to elevated levels of Tp53-induced apoptosis. This observation implies that both FTO and P53 play crucial roles in the regulatory mechanisms governing granulosa cells within follicles. Furthermore, HYF was found to reverse this outcome. This aroused our strong interest, given FTO is a demethylase of N6-methyladenosine (m6A) in RNA, elevated levels of FTO in VCD-treated cells would result in decreased m6A-Tp53 transcripts and reduced P53 levels. Subsequently, we investigated whether the knockdown of FTO could lead to the derepression of P53 expression. The results obtained align with those observed *in vivo*. Unfortunately, we do not have quantitative data or methylation site detection for m6A modifications to TP53 in FTO knockdown cell lines, thus we cannot determine the regulatory role of FTO in tp53 m6A modification, but we suggest that RNA-binding proteins, including m6A reader proteins, may play a role.

Consequently, it is essential to undertake further research to examine the potential modulation of P53 gene and protein expression in granulosa cells, specifically through the investigation of FTO or m6A methylation.

Our study presents several limitations, notably the inadequate detection of the m6A methylase system. Furthermore, the investigation into the m6A reader protein, which is essential for post-modification translation, remains unaddressed. This area warrants further exploration. Future research should include experiments incorporating inhibitors and co-immunoprecipitation (Co-IP) to substantiate the correlation between proteins. Within the limitations of time and resources, the role of m6A-Tp53 in the regulation of the molecular mechanisms of HYF was initially identified and validated under current experimental conditions. However, further research is required to determine whether HYF modulates the transcription of m6A-Tp53 via FTO and to elucidate how it influences downstream signaling through modified translation. This necessitates a more comprehensive experimental design to strengthen the existing evidence.

## 5 Conclusion

**FIGURE 8:**
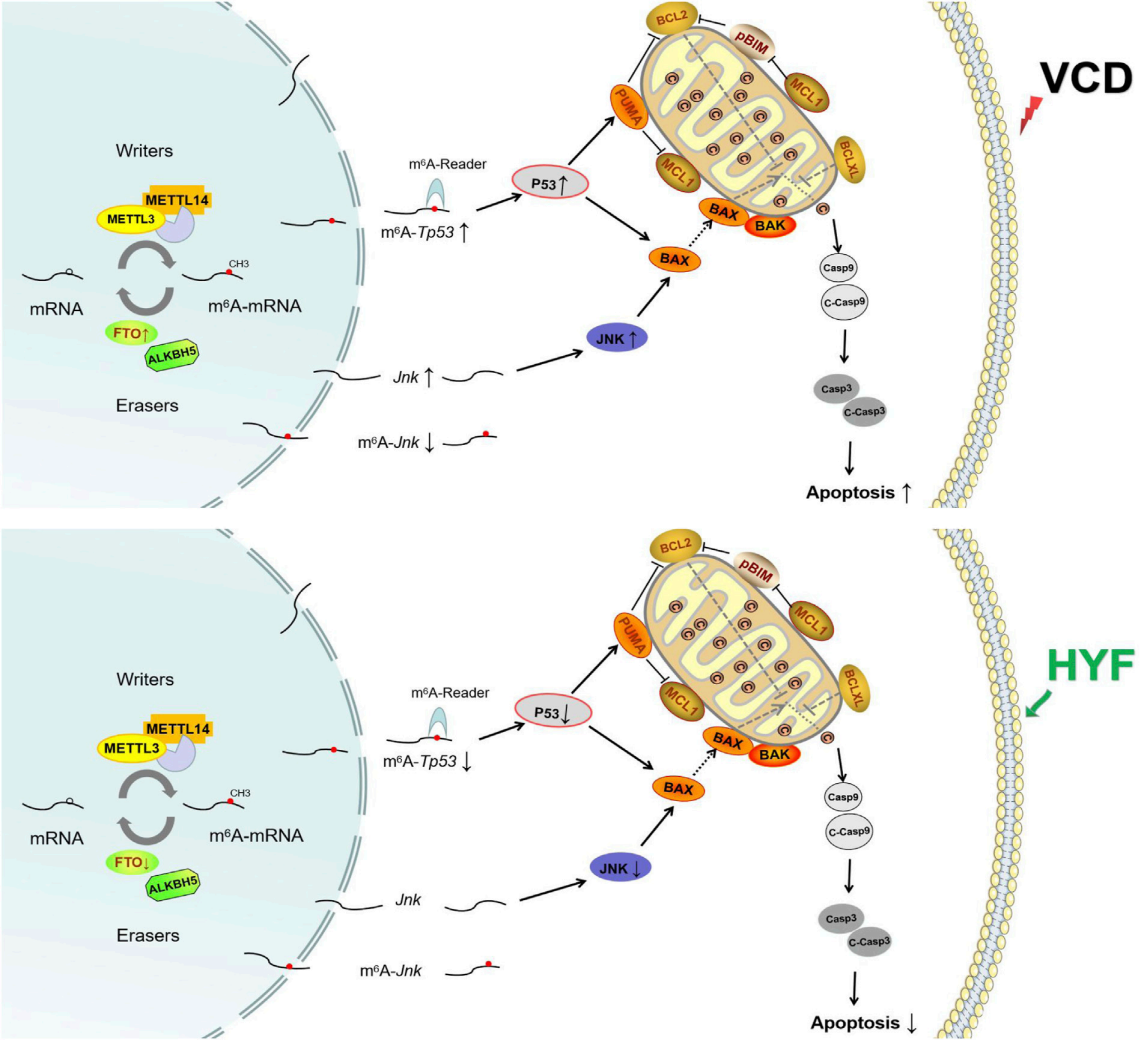
The anti-apoptotic mechanism of Huyang Yangkun formula regulates the apoptotic signaling pathway of mitochondrial P53/BCL-2 family in ovarian granulosa cells through FTO/m6A-RNA.

In conclusion, the results of this study indicate that HYF is a promising therapeutic candidate for the treatment of POI. The research utilized both *in vivo* and *in vitro* experiments to conduct an initial exploration of the dynamic regulation of m6A RNA modification influenced by HYF. These findings enhance our understanding of the molecular mechanisms through which HYF preserves ovarian function (Figure 8). Moreover, this study offers a novel examination of the molecular mechanisms by which Chinese herbal compounds protect ovarian function, specifically through the perspective of m6A RNA modification. The findings of this research hold significant implications for future studies conducted in the field of female reproduction.

## Data Availability

The original contributions presented in the study are included in the article/Supplementary Material, further inquiries can be directed to the corresponding author.

## Abbreviations

POI, premature ovarian insufficiency; VCD, 4-vinylcyclonhexene diepoxide; AMH anti-Müllerian hormone; FSH, follicle-stimulating hormone; E2, estradiol; m6A, N6-methyladenosine; METTL3, methyltransferase 3; METTL14, methyltransferase like 14; ALKBH5 alkB homolog 5; FTO, fat mass and obesity associated; JNK, c-jun N-terminal kinase; P38, mitogen-activated protein kinase14; HO1, heme oxygenase 1; NRF2, nuclearfactor (erythroid-derived2)-like2 protein; NQO1 NADPH quinine oxidoreductase1; P53 (Tp53), tumor protein p53; BCL2, B cell lymphoma 2; BCLXL, B cell lymphoma-extra large; MCL1, myeloid cell leukemia-1; BAX (Bax), BCL2-Associated X; BAK (Bak), BCL2 antagonist/killer; pBIM (phosphorylated), Bcl2 interacting mediator of cell death; PUMA (Puma), p53 upregulated modulator of apoptosis; Caspase, cysteinyl aspartate specific proteinase; MAPK, mitogen-activated protein kinase.

## References

[B1] Alvaro Mercadal B. Imbert R. Demeestere I. Gervy C. De Leener A. Englert Y. (2015). AMH mutations with reduced *in vitro* bioactivity are related to premature ovarian insufficiency. Hum. Reprod. 30, 1196–1202. 10.1093/humrep/dev042 25750103

[B2] Anderson R. A. Cameron D. Clatot F. Demeestere I. Lambertini M. Nelson S. M. (2022). Anti-Müllerian hormone as a marker of ovarian reserve and premature ovarian insufficiency in children and women with cancer: a systematic review. Hum. Reprod. Update 28, 417–434. 10.1093/humupd/dmac004 35199161 PMC9071067

[B3] Desongnis S. Robin G. Dewailly D. Pigny P. Catteau-Jonard S. (2021). AMH assessment five or more years after an initially low AMH level. Eur. J. Obstet. Gynecol. Reprod. Biol. 256, 70–74. 10.1016/j.ejogrb.2020.10.053 33171420

[B4] Ding C. Zou Q. Ding J. Ling M. Wang W. Li H. (2018). Increased N6-methyladenosine causes infertility is associated with FTO expression. J. Cell Physiol. 233, 7055–7066. 10.1002/jcp.26507 29384212 PMC13482491

[B5] Fu Y. Ding D. N. Shen Y. Jia L. Y. Yan M. Y. Wei W. (2022). Complementary and alternative medicine for premature ovarian insufficiency: a review of utilization and mechanisms. Evid. Based Complement. Altern. Med. 2022, 9053930. 10.1155/2022/9053930 PMC8993576 35399635

[B6] Golezar S. Ramezani Tehrani F. Khazaei S. Ebadi A. Keshavarz Z. (2019). The global prevalence of primary ovarian insufficiency and early menopause: a meta-analysis. Climacteric 22, 403–411. 10.1080/13697137.2019.1574738 30829083

[B7] Hu Y. Ouyang Z. Sui X. Qi M. Li M. He Y. (2020). Oocyte competence is maintained by m(6)A methyltransferase KIAA1429-mediated RNA metabolism during mouse follicular development. Cell Death Differ. 27, 2468–2483. 10.1038/s41418-020-0516-1 32094512 PMC7370231

[B8] Hua W. Zhao Y. Jin X. Yu D. He J. Xie D. (2018). METTL3 promotes ovarian carcinoma growth and invasion through the regulation of AXL translation and epithelial to mesenchymal transition. Gynecol. Oncol. 151, 356–365. 10.1016/j.ygyno.2018.09.015 30249526

[B9] Huang B. Ding C. Zou Q. Wang W. Li H. (2019). Cyclophosphamide regulates N6-methyladenosine and m6A RNA enzyme levels in human granulosa cells and in ovaries of a premature ovarian aging mouse model. Front. Endocrinol. (Lausanne) 10, 415. 10.3389/fendo.2019.00415 31316467 PMC6610338

[B10] Jiang Z. X. Wang Y. N. Li Z. Y. Dai Z. H. He Y. Chu K. (2021). The m6A mRNA demethylase FTO in granulosa cells retards FOS-dependent ovarian aging. Cell Death Dis. 12, 744. 10.1038/s41419-021-04016-9 34315853 PMC8316443

[B11] Jiao X. Meng T. Zhai Y. Zhao L. Luo W. Liu P. (2021). Ovarian reserve markers in premature ovarian insufficiency: within different clinical stages and different etiologies. Front. Endocrinol. (Lausanne) 12, 601752. 10.3389/fendo.2021.601752 33815272 PMC8015703

[B12] Ke H. Tang S. Guo T. Hou D. Jiao X. Li S. (2023). Landscape of pathogenic mutations in premature ovarian insufficiency. Nat. Med. 29, 483–492. 10.1038/s41591-022-02194-3 36732629 PMC9941050

[B13] Lence T. Akhtar J. Bayer M. Schmid K. Spindler L. Ho C. H. (2016). m(6)A modulates neuronal functions and sex determination in Drosophila. Nature 540, 242–247. 10.1038/nature20568 27919077

[B14] Li M. Xiao Y. B. Wei L. Liu Q. Liu P. Y. Yao J. F. (2022). Beneficial effects of traditional Chinese medicine in the treatment of premature ovarian failure. Evid. Based Complement. Altern. Med. 2022, 5413504. 10.1155/2022/5413504 PMC9719426 36471694

[B15] Li M. Xie L. Li Y. Liu J. Nie G. Yang H. (2020). Synergistic effect of Huyang Yangkun Formula and embryonic stem cells on 4-vinylcyclohexene diepoxide induced premature ovarian insufficiency in mice. Chin. Med. 15, 83. 10.1186/s13020-020-00362-6 32774448 PMC7405416

[B16] Li M. Zhu Y. Wei J. Chen L. Chen S. Lai D. (2023a). The global prevalence of premature ovarian insufficiency: a systematic review and meta-analysis. Climacteric 26, 95–102. 10.1080/13697137.2022.2153033 36519275

[B17] Li Y. Li M. Liu J. Nie G. Yang H. (2023b). Altered m6A modification is involved YAP-mediated apoptosis response in 4-vinylcyclohexene diepoxide induced ovotoxicity. Ecotoxicol. Environ. Saf. 262, 115192. 10.1016/j.ecoenv.2023.115192 37393819

[B18] Liu C. Li L. Yang B. Zhao Y. Dong X. Zhu L. (2022). Transcriptome-wide N6-methyladenine methylation in granulosa cells of women with decreased ovarian reserve. BMC Genomics 23, 240. 10.1186/s12864-022-08462-3 35346019 PMC8961905

[B19] Liu F. X. Sun Y. (2023). Identification of the active ingredients and pharmacological effects of Kuna capsules in the treatment of primary ovarian insufficiency: a review. Med. Baltim. 102, e33884. 10.1097/MD.0000000000033884 PMC10219746 37233423

[B20] Liu W. Chen M. Liu C. Wang L. Wei H. Zhang R. (2023). Epg5 deficiency leads to primary ovarian insufficiency due to WT1 accumulation in mouse granulosa cells. Autophagy 19, 644–659. 10.1080/15548627.2022.2094671 35786405 PMC9851269

[B21] Meulmeester E. Jochemsen A. G. (2008). p53: a guide to apoptosis. Curr. Cancer Drug Targets 8, 87–97. 10.2174/156800908783769337 18336191

[B22] Nam E. Y. Kim S. A. Kim H. Kim S. -H. Han J. H. Lee J. H. (2016). Akt activation by Evodiae Fructus extract protects ovary against 4-vinylcyclohexene diepoxide-induced ototoxicity. J. Ethnopharmacol. 194, 733–739. 10.1016/j.jep.2016.10.048 27769945

[B23] Nelson S. M. Davis S. R. Kalantaridou S. Lumsden M. A. Panay N. Anderson R. A. (2023). Anti-Müllerian hormone for the diagnosis and prediction of menopause: a systematic review. Hum. Reprod. Update 29, 327–346. 10.1093/humupd/dmac045 36651193 PMC10152172

[B24] Olvera-Juárez E. Silva C. C. Flores A. Arrieta-Cruz I. Mendoza-Garcés L. Martínez-Coria H. (2020). The content of gonadotropin-releasing hormone (GnRH), kisspeptin, and estrogen receptors (ERα/ERβ) in the anteromedial hypothalamus displays daily variations throughout the rat estrous cycle. Cell Tissue Res. 381, 451–460. 10.1007/s00441-020-03258-x 32710274

[B25] Sun X. Lu J. Li H. Huang B. (2022). The role of m(6)A on female reproduction and fertility: from gonad development to ovarian aging. Front. Cell Dev. Biol. 10, 884295. 10.3389/fcell.2022.884295 35712673 PMC9197073

[B26] Wang L. Li M. Liu J. Nie G. Li Y. Yang H. (2021). Protective effect of Huyang Yangkun Formula on ovarian function in premature ovarian insufficiency rats based on apoptotic mechanism. J. Ethnopharmacol. 280, 114477. 10.1016/j.jep.2021.114477 34343645

[B27] Welt C. K. (2008). Primary ovarian insufficiency: a more accurate term for premature ovarian failure. Clin. Endocrinol. (Oxf) 68, 499–509. 10.1111/j.1365-2265.2007.03073.x 17970776

[B28] Wu X. Deng Y. (2002). Bax and BH3-domain-only proteins in p53-mediated apoptosis. Front. Biosci. 7, d151–d156. 10.2741/A772 11779719

[B29] Yafang L. H. Y. (2018). Study on the treatment of premanture ovarian insufficiency by hu yang yang kun recipe. Guangzhou: Guangzhou University of Chinese Medicine, 16–29.

[B30] Yang H. Xie Y. Yang D. Ren D. (2017). Oxidative stress-induced apoptosis in granulosa cells involves JNK, p53 and Puma. Oncotarget 8, 25310–25322. 10.18632/oncotarget.15813 28445976 PMC5421932

[B31] Yao Y. Yang Y. Guo W. Xu L. You M. Zhang Y. C. (2021). METTL3-dependent m(6)A modification programs T follicular helper cell differentiation. Nat. Commun. 12, 1333. 10.1038/s41467-021-21594-6 33637761 PMC7910450

