# Supplementary material for: Huyang Yangkun formula regulates the mitochondria pathway of ovarian granulosa cell apoptosis through FTO/m6A-P53 pathway
Source: Front Pharmacol. 2024 Nov 8;15:1491546. doi: 10.3389/fphar.2024.1491546 (PMC11581872; doi:10.3389/fphar.2024.1491546)
Supplement: Supplementary file 1 [file Table1.docx]

**Supplementary material**

**Table 1. The sequences of the primers**

| Gene | primers |
| --- | --- |
| *Tp53* | Primer F: GTCACCTCCACACCTCCACCTG  Primer R: GGCACAAACACGAACCTCAAAGC |
| *Jnk* | Primer F: CCACCACCAAAGATCCCTGACAAG  Primer R: TCATCTACAGCAGCCCAGAGGTC |
| *Puma* | Primer F: ACCTCAACGCGCAGTACGA  Primer R: CTAGTTGGGCTCCATTTCTGG |
| *Bax* | Primer F: AGGCGAATTGGCGATGAACTGG  Primer R: TGGTGAGTGAGGCAGTGAGGAC |
| *Bak* | Primer F: AGAGTTGCCCAGGACACAGAGGAG  Primer R: ACGGTAGCCAAAGCCCAGGAG |
| *β-actin* | Primer F: CGTAAAGACCTCTATGCCAACA  Primer R: TAGGAGCCAGGGCAGTAATC |
| *Gapdh* | Primer F: ATGGCTACAGCAACAGGGT  Primer R: TTATGGGGTCTGGGATGG |

**Supplementary material**

**Table 2. Disturbance of estrous cycle in rats（n=7）**

| Group | Day 30  Disorder/normal（n） | | Day 60  Disorder/normal（n） | | Day 100  Disorder/normal（n） |
| --- | --- | --- | --- | --- | --- |
| CON | 0/7 | 0/7 | | 0/7 | |
| VCD | 4/3 | 4/3 | | 7/0 | |
| HYF | 6/1 | 3/4 | | 2/5 | |

**Supplementary material**

**Table 3. m6A modification prediction site on Tp53**

| No. | Sequence context | Score(combined) | Decision |
| --- | --- | --- | --- |
| 1 | GGGGA GCCCA CCUCA GAAAC UGACU UUGCU CUUGU AGAAG UGACC | 0.690 | m6A site (Very high confidence) |
| 2 | ----- ----- ---GG GAUUG GGACU UUCCC CUCCC ACGUG CUCAC | 0.650 | m6A site (High confidence) |
| 3 | UAAGG GGGAG CCCAC CUCAG AAACU GACUU UGCUC UUGUA GAAGU | 0.648 | m6A site (High confidence) |
| 4 | ----- ----U GCUGG GAUUG GGACU UUCCC CUCCC ACGUG CUCAC | 0.642 | m6A site (High confidence) |
| 5 | UCUGA AGCUC CAGUU CAUUG GGACU UAUCC UUGCU AUAGG UAGCG | 0.610 | m6A site (Moderate confidence) |
| 6 | GUUGG GGAGC UGUCC CAAGG GGACA GGUGA GACAC UGAUG GGGCC | 0.608 | m6A site (Moderate confidence) |
| 7 | UGUUG GAGAU UGGCU GGCUG UGACU GAAUG UCUAA GGAGC UAUGG | 0.597 | m6A site (Moderate confidence) |
| 8 | UCCCC AGAGG GAGGG GAGUA GAACU GUAGG GUGCA GAUGA GAAGG | 0.590 | m6A site (Moderate confidence) |
| 9 | UUGCC CGGAG CUGCC CCCAG GGACA CUGCC CACCA GCACA AGCUC | 0.575 | m6A site (Low confidence) |
| 10 | CCUGG GCUUC CUGCA  GUCAG GGACA AUCCG  UGGGC GUGAG CGCUU | 0.573 | m6A site (Low confidence) |
| 11 | UCCUG CAGCA CAGGA ACCUG GAACU GAGGC CCCUG CACCC GUGGC | 0.572 | m6A site (Low confidence) |
| 12 | CUGUC CUGGG AGAGA CCGUC GGACA GAGGA AGAAA AUUUC CGCAA | 0.569 | m6A site (Low confidence) |
| 13 | AGGGU GUCAC GCUCC CCUGA AGACU GGAUA ACUGU CAUGG AGGAU | 0.565 | m6A site (Low confidence) |

"Sequence context "refers to mRNA sequence, where blue is RRACH structure motif and underline" A "is m6A modification site. "Score(combined)" is a comprehensive score; "Decision" is the level of confidence.
