# Supplementary figures and images for: Huyang Yangkun formula regulates the mitochondria pathway of ovarian granulosa cell apoptosis through FTO/m6A-P53 pathway
Source: Front Pharmacol. 2024 Nov 8;15:1491546. doi: 10.3389/fphar.2024.1491546 (PMC11581872; doi:10.3389/fphar.2024.1491546)

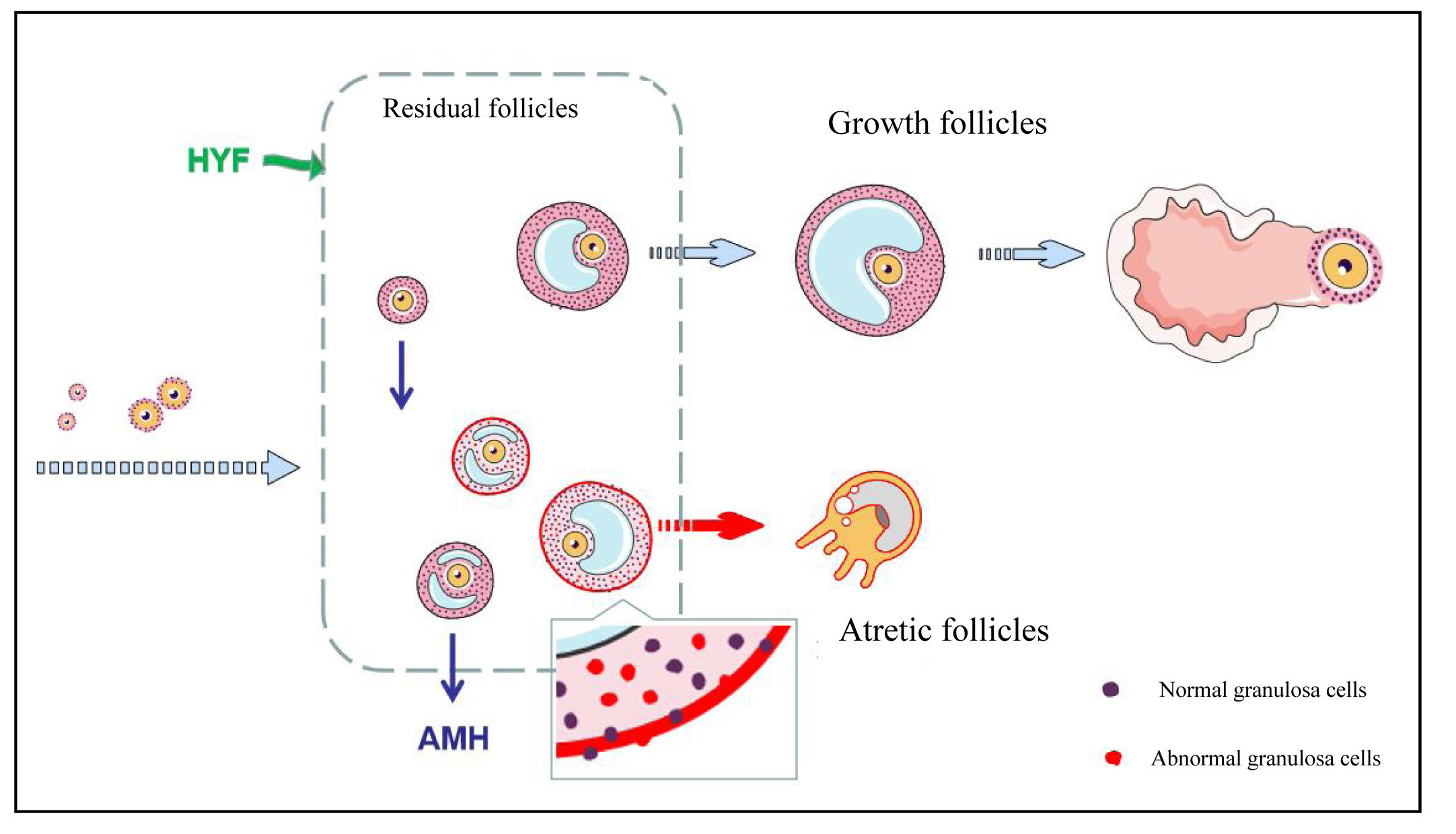

Supplement: Supplementary file 2 [file Image3.TIF]

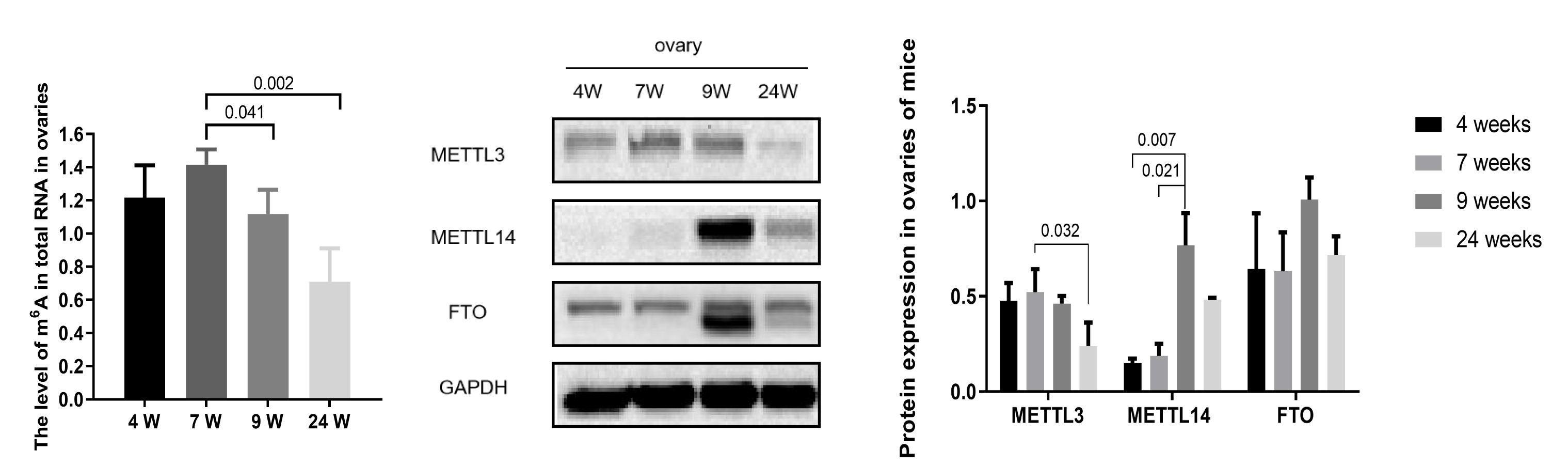

Supplement: Supplementary file 3 [file Image4.TIF]

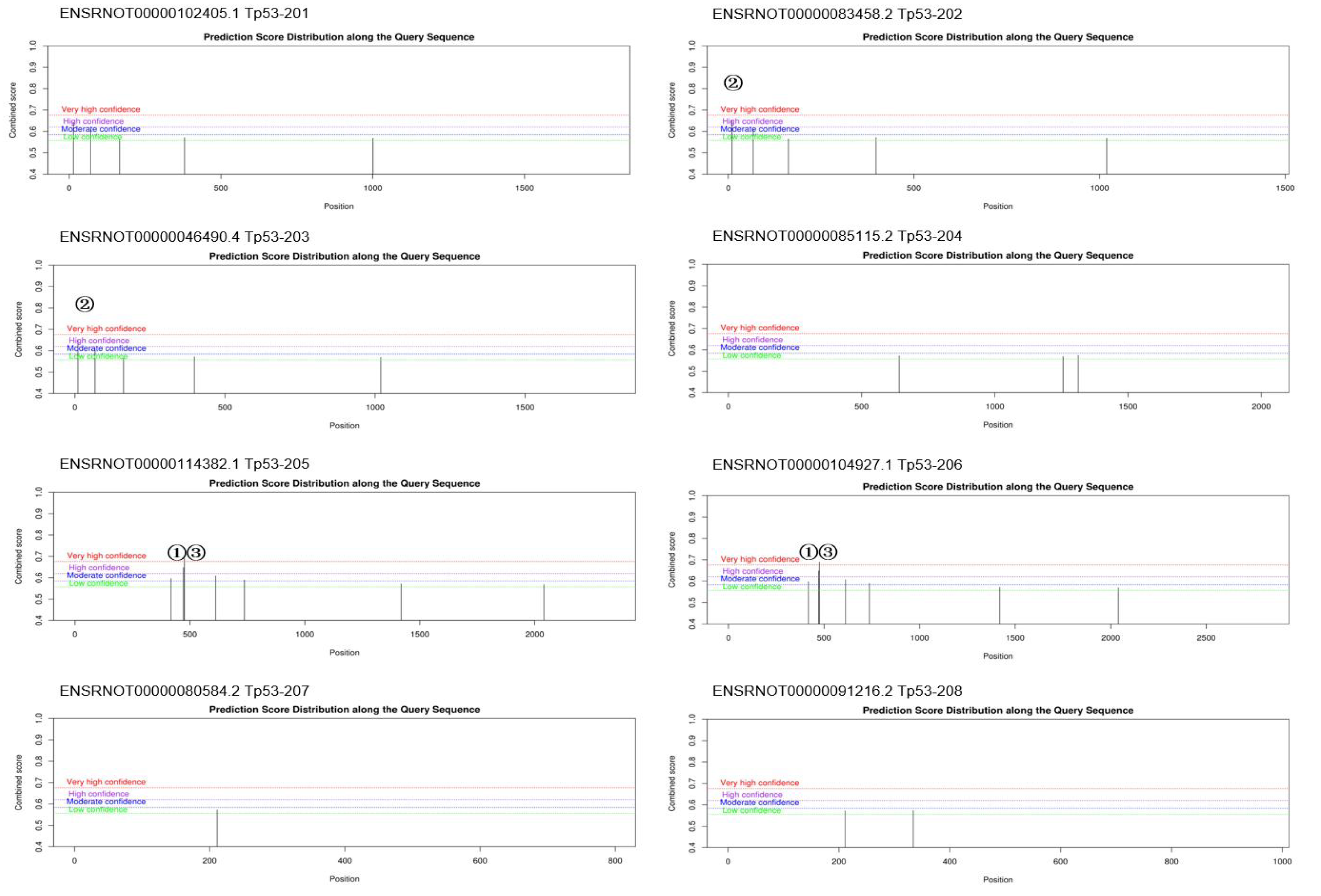

Supplement: Supplementary file 4 [file Image2.TIF]

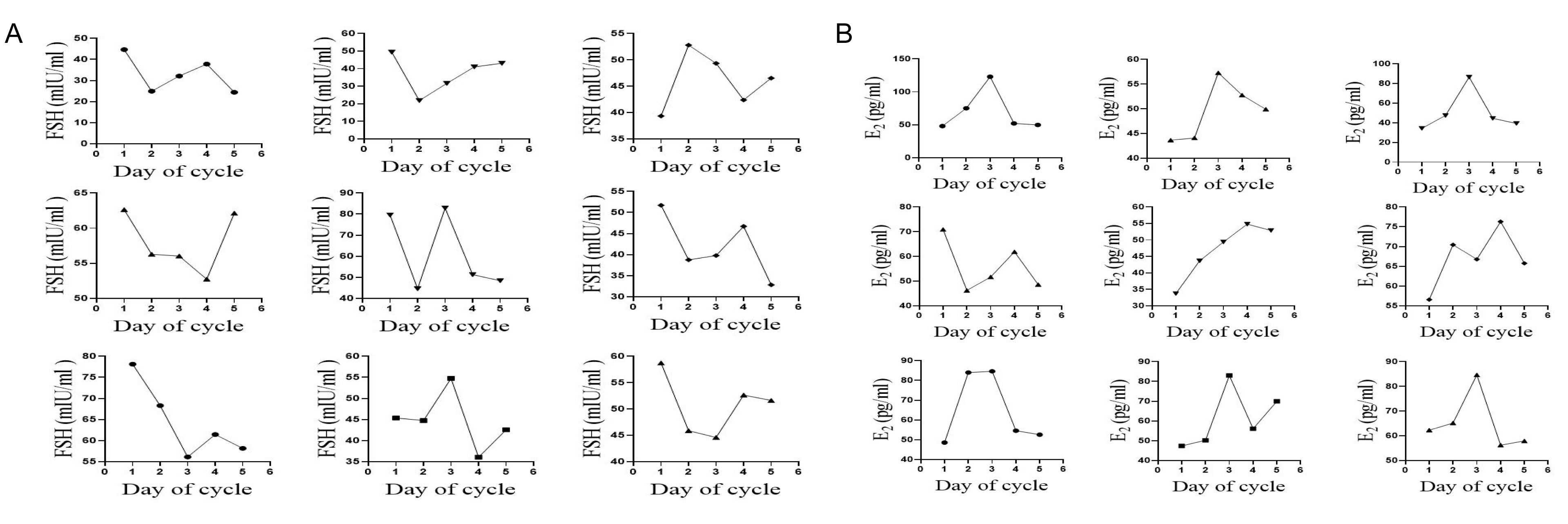

Supplement: Supplementary file 5 [file Image1.TIF]
